# Gene Expression Profiling of Glioblastoma to Recognize Potential Biomarker Candidates

**DOI:** 10.3389/fgene.2022.832742

**Published:** 2022-04-27

**Authors:** Qiang Li, S. Aishwarya, Ji-Ping Li, Dong-Xiao Pan, Jia-Pei Shi

**Affiliations:** ^1^ Department of Neurosurgery, Hwa Mei Hospital, University of Chinese Academy of Sciences (Ningbo No. 2 Hospital), Ningbo Institute of Life and Health Industry, University of Chinese Academy of Sciences, Ningbo, China; ^2^ Department of Bioinformatics, Stella Maris College (Autonomous), Chennai, India; ^3^ Department of Radiology, Hwa Mei Hospital, University of Chinese Academy of Sciences (Ningbo No. 2 Hospital), Ningbo Institute of Life and Health Industry, University of Chinese Academy of Sciences, Ningbo, China

**Keywords:** glioblastoma, hub genes, survival analysis, biomarkers, gene ontology, gene expression

## Abstract

Glioblastoma is an aggressive malignant tumor of the brain and spinal cord. Due to the blood–brain barrier, the accessibility of its treatments still remains significantly challenging. Unfortunately, the recurrence rates of glioblastoma upon surgery are very high too. Hence, understanding the molecular drivers of disease progression is valuable. In this study, we aimed to investigate the molecular drivers responsible for glioblastoma progression and identify valid biomarkers. Three microarray expression profiles GSE90604, GSE50601, and GSE134470 containing healthy and glioblastoma-affected samples revealed overlapping differentially expressed genes (DEGs). The interrelational pathway enrichment analysis elucidated the halt of cell cycle checkpoints and activation of signaling pathways and led to the identification of 6 predominant hub genes. Validation of hub genes in comparison with The Cancer Genome Atlas datasets identified the potential biomarkers of glioblastoma. The study evaluated two significantly upregulated genes, *SPARC* (secreted protein acidic and rich in cysteine) and *VIM* (vimentin) for glioblastoma. The genes *CACNA1E* (calcium voltage-gated channel subunit alpha1 e), *SH3GL2* (SH3 domain-containing *GRB2-*like 2, endophilin A1), and *DDN* (dendrin) were identified as under-expressed genes as compared to the normal and pan-cancer tissues along with prominent putative prognostic biomarker potentials. The genes *DDN* and *SH3GL2* were found to be upregulated in the proneural subtype, while *CACNA1E* in the mesenchymal subtype of glioblastoma exhibits good prognostic potential. The mutational analysis also revealed the benign, possibly, and probably damaging substitution mutations. The correlation between the DEG and survival in glioblastoma was evaluated using the Kaplan–Meier plots, and *VIM* had a greater life expectancy of 60.25 months. Overall, this study identified key candidate genes that might serve as predictive biomarkers for glioblastoma.

## Introduction

Glioma is the primary malignant brain tumor affecting the glial cells of the central nervous system (Mamelak & Jacoby, 2007). There are multiple types of glioma known based on the cells from which it originates. The main types are glioblastoma, astrocytoma, mixed gliomas, anaplastic astrocytoma, ependymomas, and oligodendroglioma. Among these, the grade IV malignant tumor glioblastoma (GBM) is the most dangerous, with a survival rate of merely 3%–5%. Unfortunately, the occurrence of the disease in the brain makes it much more difficult for early-stage diagnosis (Müller Bark et al., 2020). In general, a patient affected by GBM survives up to 5 months. However, modern surgery, radiation, and chemotherapy can extend the median survival rate to approximately 12 months (Banu, 2019). Due to the presence of endothelial cell luminal and abluminal plasma membranes, the blood–brain barrier offers physical and biochemical barriers to the normal brain and prevents the passage of oncologic drugs, lipophilic molecules, and monoclonal antibodies (Sarkaria et al., 2018). GBM causes vasogenic brain edema that results in intracranial pressure, which eventually leads to the induction of leakage and the disruption of the normal blood–brain barrier in most patients. The major challenge in prognosis, therapy, and treatments of GBM is due to their invasive nature and the inaccessibility of the brain tissues caused by the disrupted blood–brain barrier (Dubois et al., 2014).

In 2005, the Food and Drug Administration (FDA) approved standard-of-care temozolomide (Chang et al., 2004; Dubois et al., 2014) to treat newly diagnosed brain tumors. In 2009, anti-vascular endothelial growth factor (VEGF) was approved to treat GBM (Friedman et al., 2009; Kreisl et al., 2009). Researchers have recently identified targeting the rat sarcoma virus (RAS) and tyrosine kinase signaling pathways, ephrin receptor subfamily of the protein-tyrosine kinase family (EPH3A) and *EGFR* receptors, and the development of monoclonal antibody bevacizumab as the means to halt the progression of GBM (Mao et al., 2012; Pearson & Regad, 2017). Unfortunately, the recurrence of GBM tumors remains a major limiting factor for all the existing primary treatment strategies (Shergalis et al., 2018; Cheng et al., 2021). Thus, there is an unmet need to identify molecular drivers responsible for the progression of GBM.

In this direction, discerning the key aberrant molecular targets and pathways in the initiation and progression of GBM can be a significant strategy for developing potential therapeutics (Rai & Jamil, 2019; Silantyev et al., 2019). In previous studies, the genomic, transcriptomic, and proteomic profiles of the healthy and diseased samples have been widely used in elucidating the pathogenesis of cancer and other diseases (Tang & Zhang, 2018; Hsu et al., 2019; Yin et al., 2019). One such recent study elucidated ADAM-like decysin 1 (ADAMDEC1) and fibroblast growth factor 2 (FGF2) as novel druggable targets for GBM (Jimenez-Pascual et al., 2019).

In the current study, we have analyzed the microarray gene expression profiles of GBM collected from three different cohort studies. The microarray expression profile datasets, GSE90604, GSE134470, and GSE50161, were collected from the NCBI-Gene Expression Omnibus database (NCBI-GEO), and the differentially expressed genes (DEGs) with log2FC > 1 and *p-*value <0.05 were identified. It has been advocated that the integration and reanalysis of the genomics profiles from different studies offer better solutions to the poor reproducibility of single cohort studies and help ensure consistency in the analysis (Bo et al., 2018; Li et al., 2020; Yan et al., 2019). The identified DEGs were further subjected to Gene Ontology (GO) analysis to understand the potentially significant genes. The inter-relational pathways involved in the pathology of GBM were also examined to identify potential prognostic biomarkers. Additionally, the hub genes in GBM were also identified and validated through the analysis of their expression patterns in normal tissues and other related cancers. Furthermore, they were also classified based on the GBM subtypes, and the significant mutations were predicted. Additionally, the correlation between the DEG expression and survival in GBM was analyzed.

## Methodology

### Identification of Differentially Expressed Genes

The Gene Expression Omnibus (GEO) database contained gene expression profiles of GBM data from which three publicly available datasets, GSE90604 (Gulluoglu et al., 2018), GSE50161 (Griesinger et al., 2013), and GSE134470 (Golebiewska et al., 2020) were retrieved (Table 1). The platform for GSE90604 was based on the GPL17692 Affymetrix human gene 2.1 ST array with 25 samples of mRNA expression datasets from GBM and control. The platform for GSE50161 was based on the GPL570 Affymetrix human genome U133 Plus 2.0 array, and that for GSE134470 was based on the GPL6244 Affymetrix human gene 1.0 ST array expression profiling. The three aforementioned datasets were chosen for the following two reasons: 1) they are from human GBM tissues and 2) the samples were devoid of any treatment options. The overlapping differentially expressed genes were discerned through a meta-analysis of all three datasets using the limma package and Bioconductor GEO2R package. Standard data processing and analysis were implied to identify the DEGs. The datasets were standardized and log-transformed, and Benjamini–Hochberg false discovery rate statistics were applied with log2FC > 1 and *p-*value <0.05 thresholds. The bioinfokit (https://pypi.org/project/bioinfokit/) package implemented in Python was used to create the volcano plot of the DEGs. The protocol and the data files used in the study are made available for access at https://github.com/aishwarya-sekar/Glioma-gene-expression-analysis.git.

**TABLE 1:** Datasets used in the current study.

GEO accession	Platform	Type of samples	Samples available in the dataset	Samples chosen for the study
GSE90604	GPL17692 Affymetrix human gene 2.1 ST array	Expression data from GBM patient tumor samples, healthy brain tissue (partly from GBM patients) and NHA cell line and human fetal astrocyte cell line mRNA expression dataset	7 healthy tissues, 16 GBM tissues, and 2 fetal human astrocytes cell lines	7 healthy brain tissues and 16 GBM tissues
	GPL21572 Affymetrix multispecies miRNA-4 array	Expression data from GBM patient tumor samples, healthy brain tissue (partly from GBM patients) and NHA cell line and human fetal astrocyte cell line miRNA expression dataset	7 healthy tissues, 16 GBM tissues, and 2 fetal human astrocytes cell lines	Nil
GSE50601	GPL570 Affymetrix human genome U133 plus 2.0 array	Expression data from human brain tumors and human normal brains	15 Pilocytic astrocytoma (PA), 44 ependymoma (EPN), 32 glioblastomas (GBM), 22 medulloblastomas (MED), and 13 non-tumor brain (NT) control samples	32 GBM and 13 NT control samples
GSE134470	GPL6244 Affymetrix human gene 1.0 ST array	Gene expression analysis reveals a close resemblance between glioblastoma (GBM) patient tumors and corresponding patient-derived orthotopic xenografts (PDOXs)—58 samples	6 human GBM tissues, 2 normal brain tissues, 6 GBM patient-derived organoids, 5 GBM cell lines, and 5 GBM derived xenografts	6 human GBM tissues and 2 normal human brain tissues

### Gene Ontology Term and Signaling Pathway Enrichment Analysis

The DEGs were annotated for their gene ontology terms using the Database for Annotation, Visualization, and Integrated Discovery (DAVID) tool (https://david-d.ncifcrf.gov/) (Dennis et al., 2003). Pathway analysis was performed using the metabolic pathway databases REACTOME (http://www.reactome.org) (Croft et al., 2011) and KEGG (www.genome.jp/kegg) (Kanehisa & Goto, 2000). The ClueGO (version 2.5.7) module of the Cytoscape software (version 3.8.2) was used to examine the inter-relational analysis of the gene annotations and the pathway terms to predict the most significant genes. Hypergeometric two-sided tests and Benjamini–Hochberg methods were used (Bindea et al., 2009).

### Protein–Protein Interaction and Hub Gene Validation

The STRING database (http://stringdb.org/) was used to evaluate the evidence-based protein–protein interactions (PPI) (Szklarczyk et al., 2017). Active interactions based on experiments, neighborhood, co-expression, gene fusion, co-occurrence, and text mining were filtered at a medium confidence score of 0.4 and a high confidence score of 0.7. The significant PPI clusters for the up- and downregulated DEGs were determined using the MCODE plug-in implemented in the Cytoscape software. To predict the hub genes, cutoff values of 3, 2, and 0.2 were selected for the network scoring degree, K-score, and node score, respectively. The expression profiles of the hub genes were validated using the expression atlas platform (https://www.ebi.ac.uk/gxa/home) (Papatheodorou et al., 2020). The analysis based on the statistical t-tests was performed across multiple tissue levels with a maximum of 1,642 transcripts per million (TPM) expressions (Pellegrini et al., 2016) in comparison with the Cancer Genome Atlas (TCGA) GBM data from the UALCAN database (http://ualcan.path.uab.edu/cgi-bin) (Chandrashekar et al., 2017). The mutational significance of the hub genes was identified and interpreted through cBioPortal (www.cbioportal.org) with the TCGA GBM datasets (Tomczak et al., 2015). The significant substitution mutations for the hub genes were identified using the PolyPhen-2 (http://genetics.bwh.harvard.edu/pph2/bgi.shtml) webserver.

### Survival Plot Analysis and Prognostic Abilities of Hub Genes

Survival plot analysis was performed with Kaplan–Meier servers (http://kmplot.com/analysis/). It was used to predict the correlation between DEGs and the survival rates of the patients using the log-rank *p* tests. The relationships below a *p*-value of 0.05 were considered significant (Mishra et al., 2019). The molecular subtypes of GBM were identified as proneural, classical, and mesenchymal (Sidaway, 2017). The expression levels of hub genes were evaluated using the Glioblastoma BioDiscovery Portal (GBM-BioDP) (https://gbm-biodp.nci.nih.gov) to identify their prognostic ability based on the GBM subtypes (Verhaak et al., 2010) from the gene expression datasets of Verhaak 840 Core, integrated with three microarray platforms.

### Identification of Prognostic Biomarkers Through Pan-Cancer Analysis

The association of the hub genes and survival analysis revealed the predominant biomarkers of GBM. The genes were analyzed for their expression rates across multiple cancers to identify a reliable biomarker. The pan-cancer analysis was performed using the UALCAN database and protein atlas platform (Sasmita et al., 2018). The genes that had a predominant alteration in the related disease states of glioma were evaluated, and genes with significant differences in the levels of expression for GBM were identified as biomarkers of GBM.

## Results

### Differentially Expressed Gene Prediction

**FIGURE 1:**
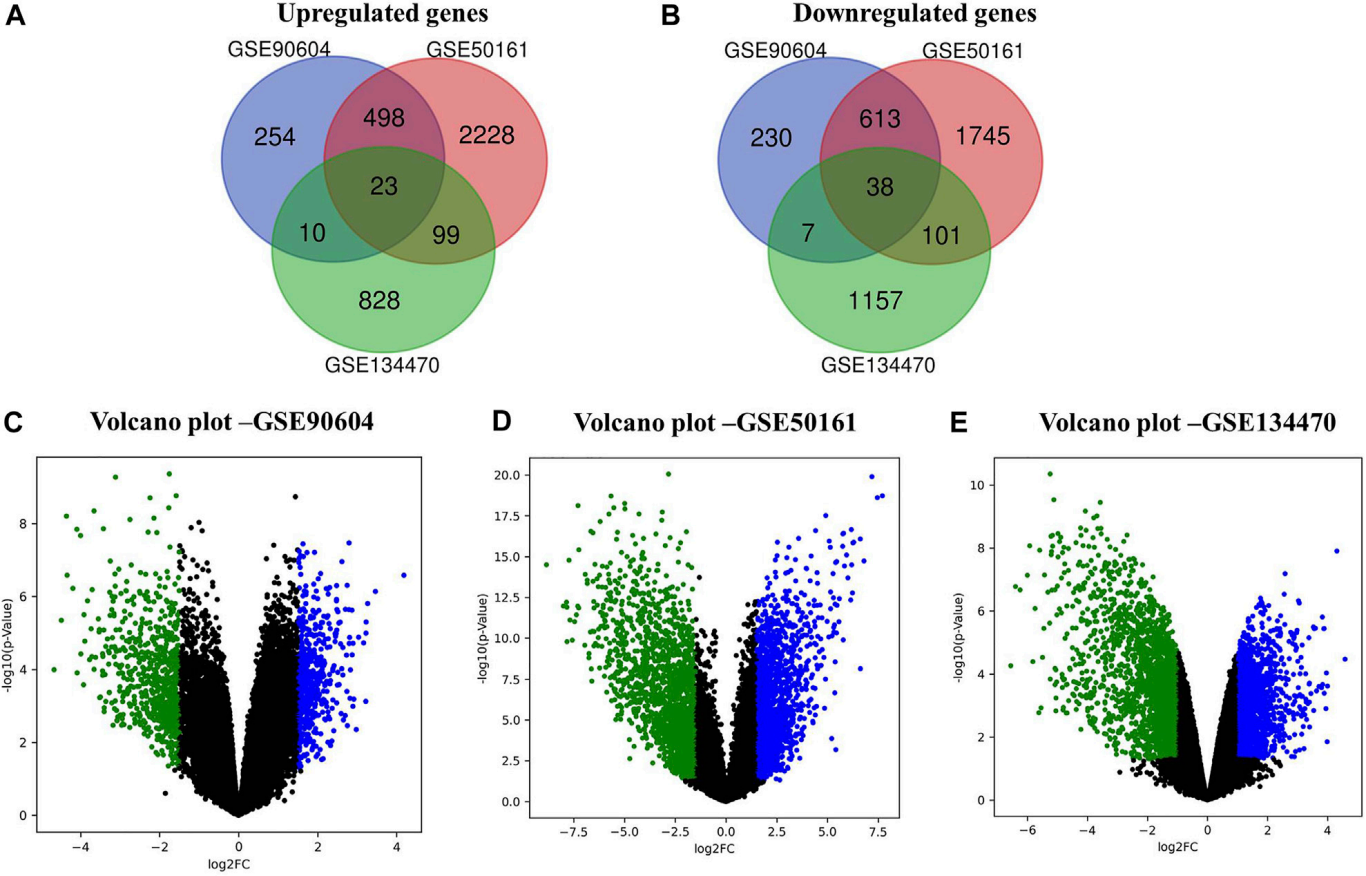
Differentially expressed genes (DEGs) of the expression profiles GSE90604, GSE50161, and GSE134470 with log2FC > 1 and *p*-value < 0.05. **(A)** Venn diagram representation of the overlapped upregulated DEGs **(B)** Venn diagram representation of the overlapped downregulated DEGs. **(C)** Volcano plots of the up- and downregulated DEGs of GSE90604. **(D)** Volcano plots of the up- and downregulated DEGs of GSE50161 and **(E)** Volcano plots of the up- and downregulated DEGs of GSE134470. Green and blue dots represent down and upregulated genes, respectively. Black dots represent the remaining genes with no significant difference.

In this work, we analyzed the microarray gene expression profiles of three GBM datasets, namely, GSE90604, GSE50161, and GSE134470, obtained from the NCBI-GEO database. The datasets were analyzed using the limma package by setting the cutoff criterion as log2FC > 1 and a *p-*value < 0.05 to obtain the DEGs. A total of 1,673 (785 upregulated and 888 downregulated), 5,375 (2,848 upregulated and 2,497 downregulated), and 2,263 (960 upregulated and 1,303 downregulated) DEGs were identified for GSE90604, GSE50161, and GSE134470, respectively. Out of these, 61 genes (23 upregulated and 38 downregulated) were found to be overlapped, as shown in Figures 1A, B). A volcano plot of all the DEGs of GSE90604, GSE50161, and GSE134470 is shown in Figures 1C–E, respectively. The overlapped DEGs (as listed in Supplementary File S1) were taken into consideration for further analysis. The topmost enriched overlapping DEGs with their biological functions are presented in Table 2.

**TABLE 2:** Top DEGs and their biological functions.

Enriched DEGs	Gene names	Biological process
Upregulated DEGs		
*CLEC7A*	C-type lectin domain-containing 7A	Regulation of dendritic cell cytokines, regulation of cell maturation and leukocyte mediated immunity
*DDX58*	DExD/H-box helicase 58	Interleukin 6 and 8 production, tumor necrosis factor production, cellular response to dsRNA, RIG-I signaling pathway
*SOCS3*	Suppressor of cytokine signaling 3	Cellular response to cytokine stimulus, interleukin-6-mediated signaling pathway
*PTPRZ1*	Protein tyrosine phosphatase receptor type Z1	Oligodendrocyte differentiation, regulation of myelination, and neural precursor cell proliferation
*MCM3*	Minichromosome maintenance complex component 3	Pre-replicative complex assembly, double-strand break repair *via* homologous recombination nuclear cell cycle DNA replication
*SEMA5A*	Semaphorin 5A	Regulation of endothelial cell migration, proliferation, axon extension involved in axon guidance and regulation of cell adhesion
*CTSK*	Cathepsin K	Chromatin modification, autophagy of mitochondrion and extracellular matrix disassembly, keratinocyte differentiation
*VIM*	Vimentin	Intermediate filament organization, cellular response to muramyl dipeptide, regulation of collagen metabolism, and glial cell differentiation
*SPARC*	Secreted protein acidic and rich in cysteine	Cell morphogenesis, regulation of endothelial cell and epithelial cell proliferation, regulation of anatomical structure morphogenesis
*HSPG2*	Heparan sulfate proteoglycan 2	Inflammatory response, angiogenesis, circulatory system development
*HELLS*	Lymphoid-specific helicase	DNA methylation, alkylation, demethylation, chromatin remodeling, centromere complex assembly, DNA metabolic process
Downregulated DEGs		
*DLG2*	Discs large MAGUK scaffold protein 2	Protein localization to presynapse, cellular response to potassium ion, axo-dendritic protein transport
*BRSK1*	BR serine/threonine kinase 1	Chemical synaptic transmission, cell morphogenesis involved in neuron differentiation, regulation of plasma membrane bounded cell projection organization, associative learning, neuron differentiation
*MAPK8IP2*	Mitogen-activated protein kinase 8 interacting protein 2	Regulation of postsynaptic membrane potential, dendrite morphogenesis, regulation of apoptotic signaling pathway, regulation of stress-activated MAPK cascade
*SHISA7*	Shisa family member 7	Regulation of neuronal synaptic plasticity
*CACNA1E*	Calcium voltage-gated channel subunit alpha1 S	Chemical synaptic transmission, anterograde trans-synaptic signaling
*DDN*	Dendrin	RNA polymerase II cis-regulatory region sequence-specific DNA binding, cognitive function, maintain bone density
*SH3GL2*	SH3 domain-containing GRB2-like 2, endophilin A1	Cellular response to brain-derived neurotrophic factor stimulus, neuron projection development, plasma membrane bounded cell projection organization, nervous system development
*SV2B*	Synaptic vesicle glycoprotein 2B	Transmembrane transporter activity, chemical synaptic transmission
*SYNJ2*	SYNJ2 intronic transcript 1	Brain development, phosphatidylinositol biosynthetic process
*PPIP5K1*	Diphosphoinositol pentakisphosphate kinase 1	Phosphate-containing compound metabolic process

### Gene Annotations From the Differentially Expressed Genes

**FIGURE 2:**
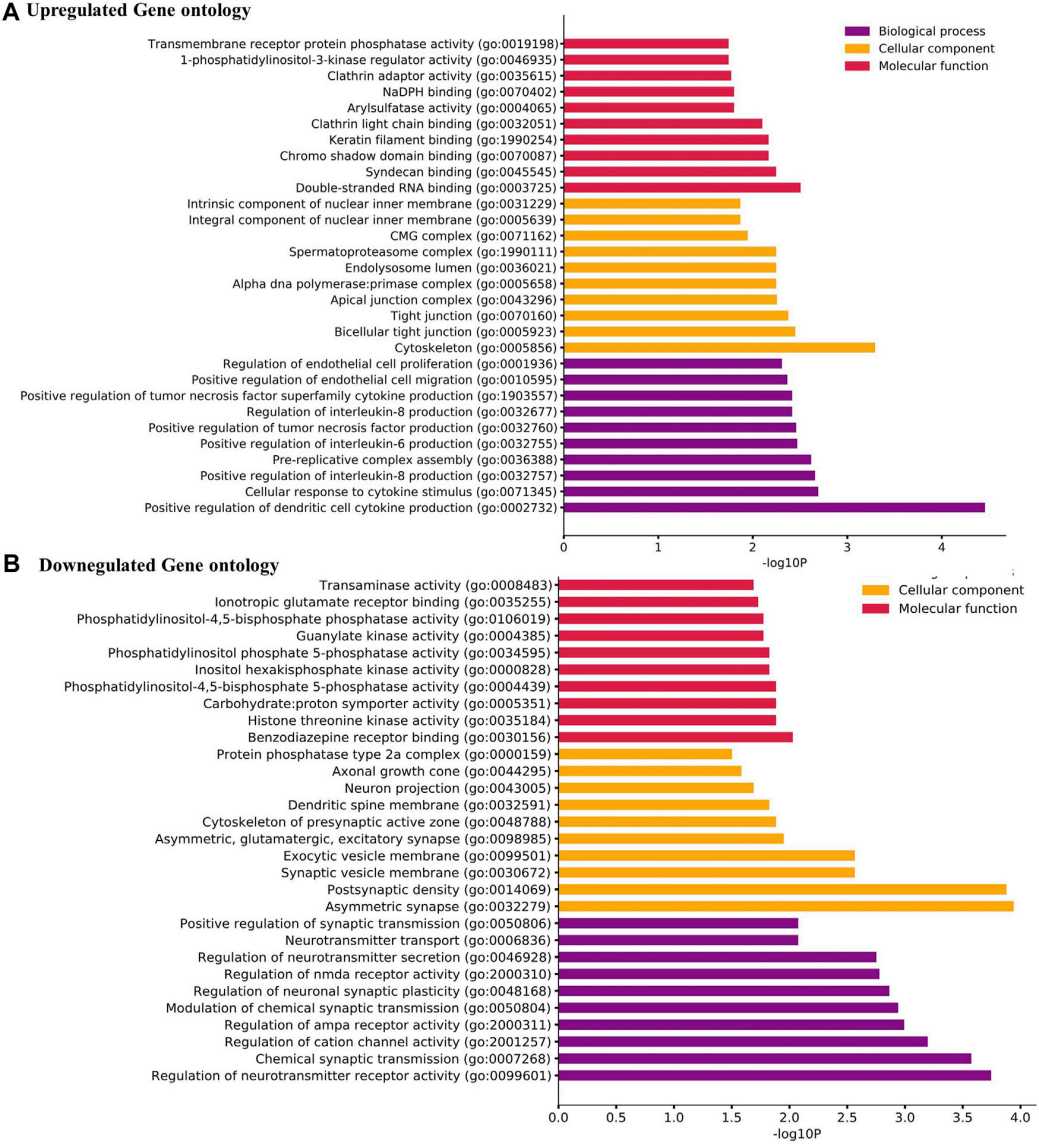
**(A)** Gene Ontology (GO) term enrichment analysis of the upregulated DEGs. **(B)** GO analysis of the downregulated DEGs. The top ten annotations ranked based on *p-*values are shown for three sub-ontologies, namely, biological process, molecular function, and cellular component.

From the GO analysis of the overlapping DEGs, the top ten annotations based on their *p-*values were considered for biological process (BP), molecular functions (MF), and cellular component (CC) sub-ontologies. As shown in Figure 2A, the upregulated genes for BP showed significant enrichment in the regulation and migration of endothelial cells, cytokine response, and regulation of interleukin-6 and interleukin-8. For MF, the upregulation in clathrin adapter activity, transaminase activity, 1-phosphatidylinositol 3 kinase regulator activity, and double-stranded RNA binding were observed (Figure 2A). For CC, the remarkable enrichment in the tight junctions, lysosomes, primase complex, and CMG complexes involved in replication was observed (Figure 2A). For BP, significant downregulation in neurotransmitter transports, synaptic transmission, cation channel activity, and neurotransmitter receptor activity was observed (Figure 2B). For CC, the downregulation in axonal and neuronal growth, exocytic vesicle membrane, and synaptic vesicle membranes were observed (Figure 2B). For MF, the downregulation in ionotropic glutamate receptor activity, guanylate activity, phosphatidylinositol 4,5 bisphosphate activity, and histone threonine kinase activity were observed (Figure 2B).

### Enrichment of Pathways

**FIGURE 3:**
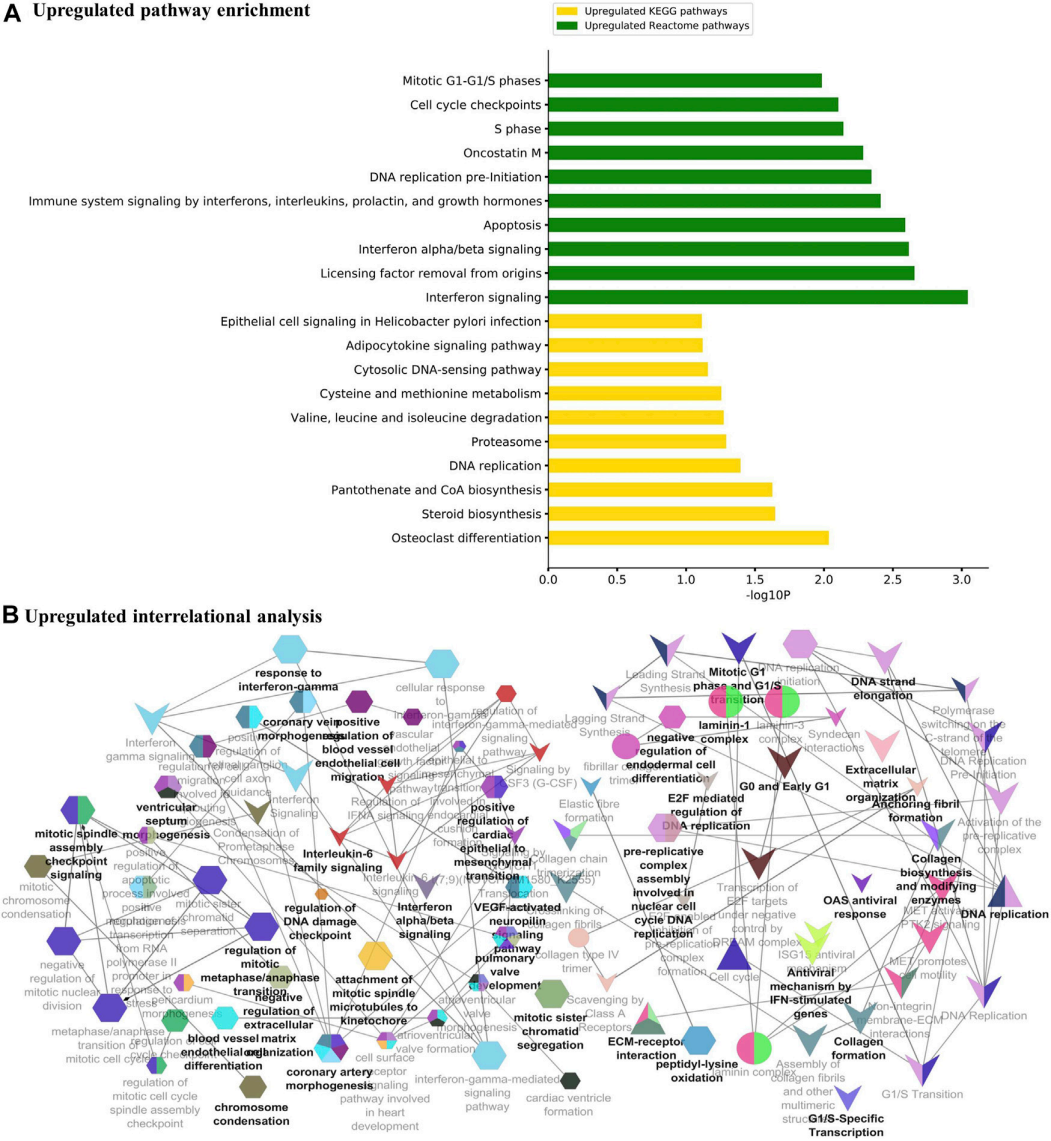
**(A)** Pathway enrichment analysis with KEGG and REACTOME is shown for the upregulated DEGs. **(B)** Interrelational pathway enrichment analysis is shown for the upregulated DEGs.

**FIGURE 4:**
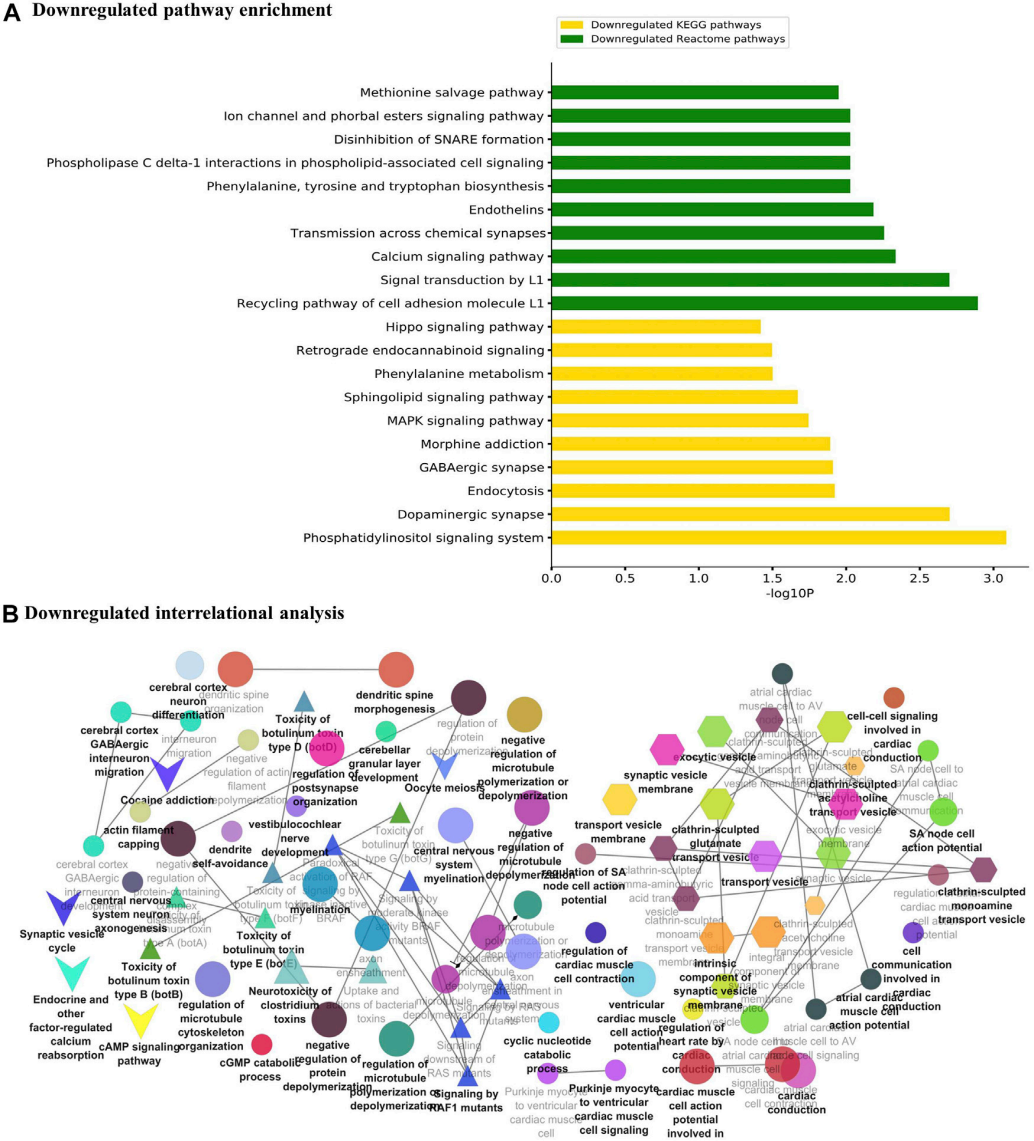
**(A)** Pathway enrichment analysis with KEGG and REACTOME is shown for the downregulated DEGs. **(B)** Interrelational pathway enrichment analysis is shown for the downregulated DEGs.

The major metabolic pathway databases, Kyoto Encyclopedia of Genes and Genomes (KEGG) and REACTOME, were used to study the enriched pathways for the annotated genes. The significant pathways involved in GBM were identified for the DEGs. As shown in Figure 3A, the upregulated DEGs were mainly involved in cell cycle checkpoints, mitotic G1-G1/S phase, DNA replication, immune system signaling of interferons, interleukins, and cytokines. Further, to ensure consistency between the GO and pathways enrichment, the inter-relational analysis was performed using the ClueGO module of Cytoscape. The upregulation of DNA replication, cell cycle checkpoints, chromosome condensation, immune responses, pulmonary valve morphogenesis, and coronary artery developments was noticed (Figure 3B). The downregulated DEGs were mainly involved in GABAergic and dopaminergic synaptic transmission, calcium signaling, osteoclast formation, SNARE formation, and methionine salvage pathways (Figure 4A). The interrelational analysis of downregulated DEGs was also consistent with the underexpression of GABA receptor activities, cell communications, synaptic transmission, and neuron myelination (Figure 4B).

### Prediction of Hub Genes Through PPI Network

**FIGURE 5:**
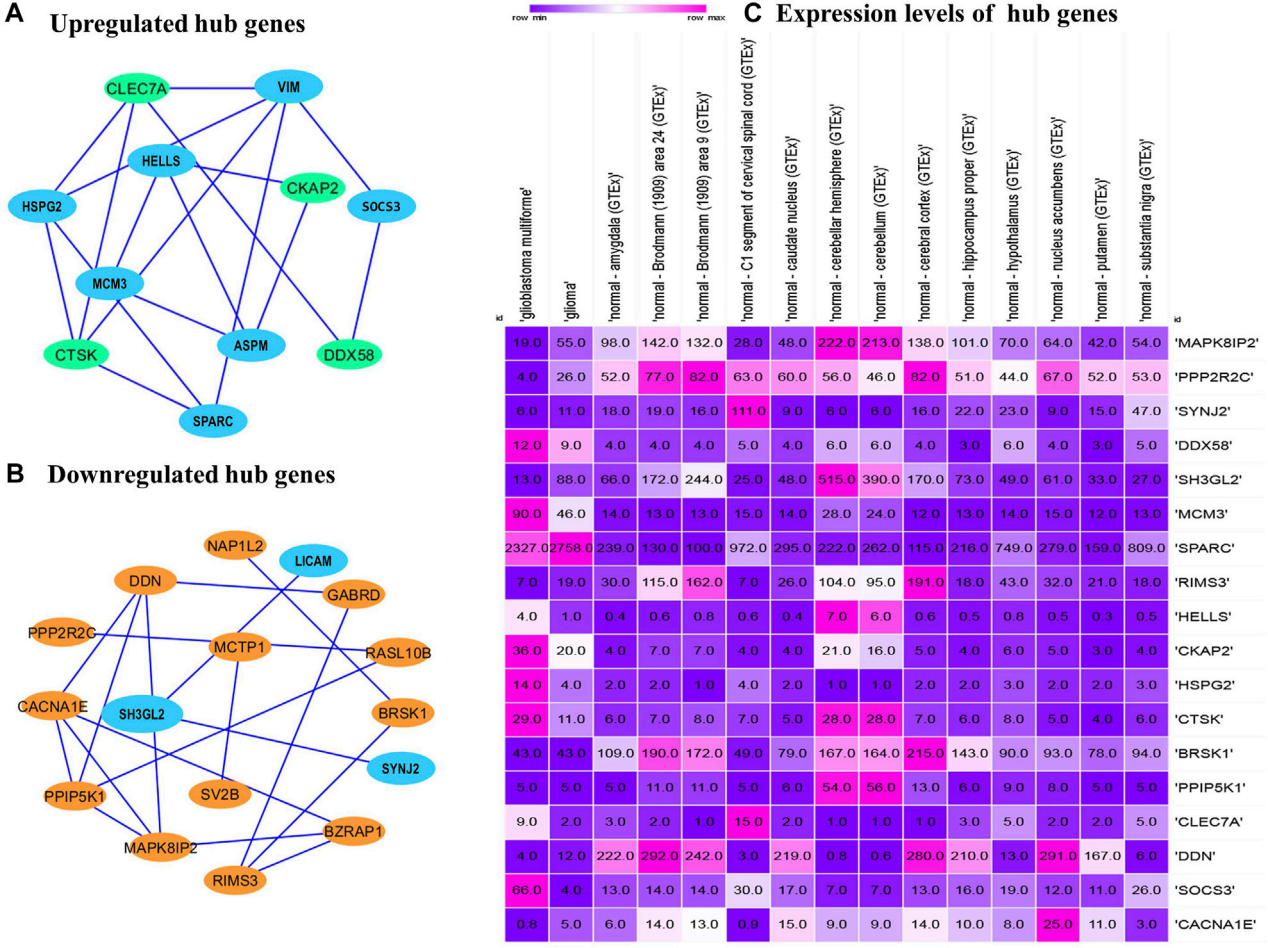
Protein–protein interaction network of the overlapped DEGs. **(A)** Upregulated hub genes of the PPI network with a medium confidence score of 0.4 are shown as green nodes, and hub genes with a high confidence score of 0.7 are shown as blue nodes. **(B)** Downregulated hub genes of the PPI with a medium score are shown as orange nodes, and hub genes with a high confidence score of 0.7 are shown as blue nodes. PPI, protein–protein interaction. DEGs, differentially expressed genes. **(C)** Comparison of the expression levels of hub genes among the brain tissues identified from the Expression Atlas platform for TCGA datasets.

The PPI networks analysis has helped in the identification of the hub genes playing a critical role in the GBM. In the predicted PPI networks for up- and downregulated DEGs, a haircut operation with a network scoring cutoff of 2 was applied using the MCODE plug-in of Cytoscape software. It resulted in 11 and 16 nodes for 23 up and 38 downregulated overlapping DEGs with scores of 3 and 3.86, respectively. The PPI networks for up- and downregulated DEGs are shown in Figures 5A, B, respectively. The 11 prominent upregulated hub genes, namely, *CTSK*, *HSPG2*, *SPARC*, *VIM*, *SOCS3*, *HELLS*, *CKAP2*, *ASPM*, *MCM3*, *DDX58*, and *CLEC7A*, were identified. A total of 16 genes, namely, *MAPK8IP2*, *CACNA1E*, *BZRAP1*, *RIMS3*, *GABRD*, *DDN*, *BRSK1*, *NAP1L2*, *PPIP5K1*, *RASL10B*, *PPP2R2C*, *SYNJ2*, *SH3GL2*, *LICAM*, *MCTP1*, and *SV2B*, were identified. Among them, seven upregulated DEGs—*VIM*, *HELLS*, *SPARC*, *HSPG2*, *MCM3*, *ASPM*, and *SOCS3* and three downregulated DEGs—*SH3GL2*, *LICAM*, and *SYNJ2* exhibited interactions with a high confidence score of 0.7.

### Validation of Hub Genes

The expression atlas platform was used to further elucidate the levels of hub genes expression on the brain tissues. The set of hub genes was validated by comparing their expression patterns against the datasets of the pan-cancer atlas of the whole-genome consortium (ICGC/TCGA Pan-Cancer Analysis of Whole Genomes Consortium, 2020). GBM is reported to occur in the regions of cerebral hemispheres and frontal and temporal lobes. As shown in Figure 5C, *MAPK8IP2*, *SH3GL3*, and *PPIP5K1* were found to be overly expressed in the tissues of the normal cerebellar hemisphere and cerebellum, whereas high expression of *PPP2R2C* and *BRSK1* was seen in the normal cerebral cortex areas of brains. A very significant expression of SPARC at the rates of 2,327.0 TPM and 2,758.0 TPM were observed in GBM and glioma, respectively. It indicates *SPARC* to be a strong prognostic biomarker of GBM and glioma. The expression levels of the genes were measured at a maximum rate of 3,642 transcripts per million. We have further extended the validation of the hub genes by comparing the expression levels with The Cancer Genome Atlas (TCGA) data. The expression levels of the hub genes in normal brains were compared to the GBM brains. They were also classified based on TP53 mutant and non-mutant tissue samples from the TCGA datasets (TCGA pan-cancer atlas with 592 samples of GBM) and shortlisted the ones that were in line with the previous comparisons, as shown in Table 3. The genes *PPP2R2C*, *DDN*, *SH3GL2*, *MAPK8IP2*, *CACNA1E*, and *BRSK1* were found significantly underexpressed, whereas *SPARC*, *VIM*, and *MCM3* were highly expressed in both the TCGA datasets (including the TP53 mutant and non-mutant types) and GEO datasets. On the contrary, the genes *CTSK*, *CKAP2*, *DDX58*, and *HSPG2* of GBM in TCGA were observed to be significantly overexpressed in comparison to their expressions in GEO datasets. The genes *SYNJ2* and *RIMS3* were observed to be relatively underexpressed in the TCGA datasets in comparison to the GEO datasets and thus excluded from further analysis.

**TABLE 3:** Expression levels of the hub genes in GBM identified from pan-cancer analysis.

Gene	Normal expression (TPM)	*p*-value	Tumor expression (TPM)	Tumor *p*-value	TP53 mutant expression (TPM)	TP53 mutant-*p*-value	TP53-non mutant expression (TPM)	TP53 non-mutant—*p-*value
*DDN-*dendrin	203.728	0.056201	1.335	0.056605	1.449	0.056879	1.21	0.56348
*SH3GL2-* SH3 domain-containing GRB2-like 2, endophilin A1	226.596	0.03476	6.651	0.032862	10.784	0.167819	5.264	0.032098
*CACNA1E*-calcium voltage-gated channel subunit alpha1 E	28.652	0.030956	0.805	0.02822	1.15	0.0274	0.72	0.0418
*PPP2R2C-*protein phosphatase 2 regulatory subunit B’gamma	185.898	0.0070628	3.91	0.0073005	3.506	0.096261	4.072	0.0074367
*MAPK8IP2-*mitogen-activated protein kinase 8 interacting protein 2	345.321	0.0134587	24.288	0.0139826	22.916	0.146618	25.11	0.0143359
*BRSK1-*BR serine/threonine kinase 1	161.358	1.62436730732907E-12	24.98	<1E-12	23.686	0.82764	4.92	<1E-12
*RIMS3-*regulating synaptic membrane exocytosis 3	96.441	0.054177	4.115	0.052856	4.611	0.48738	3.571	0.052296
*CLEC7P-*C-type lectin domain containing 7P	4.501	1.64369999999803E-06	9.959	7.7704999990047E-08	9.537	0.90746	9.801	4.78749999643924E-08
*SPARC-*secreted protein acidic and rich in cysteine	238.338	<1E-12	2055.527	1.62447832963153E-12	1,849.872	0.0162786	2,168.427	1.62436730732907E-12
*VIM-*vimentin	315.18	1.55431223447522E-15	3,474.583	1.62447832963153E-12	3,051.469	0.85376	3,571.568	1.62436730732907E-12
*MCM3-*assembly factor for spindle microtubules	14.75	1.6278089987054E-12	49.41	1.11022302462516E-16	53.018	0.0021043	45.933	9.99200722162641E-16
*SYNJ2-*synaptojanin 2	50.67	0.112542	4.872	0.114896	5.7	0.28404	4.85	0.116481
*SCOCS3-*suppressor of cytokine signaling	31.655	0.65318	45.307	0.42558	34.5	0.15806	48.45	0.36338
*PPIP5K1-*diphosphoinositol pentakisphosphate kinase 1	24.329	0.030397	5.723	0.030943	5.779	0.031338	5.674	0.031338
*CTSK-*cathepsin K	5.425	1.15290000002322E-06	20.731	1.62503344114384E-12	18.61	0.67398	20.63	1.43880463099322E-11
*CKAP2-*cytoskeleton-associated protein 2	7.45	2.4300994549975E-10	23.446	<1E-12	24.46	0.038943	21.966	7.7715611723761E-16
*DDX58-*DEAD-box helicase 58	4.18	0.00193986	6.882	0.0026363	6.947	0.8288	6.82	0.0020031
*HSPG2-*heparan sulfate proteoglycan	4.52	3.74589248508528E-13	31.03	1.62436730732907E-12	32.04	0.97406	29.68	<1E-12

### Mutational Analysis of the Hub Genes

**FIGURE 6:**
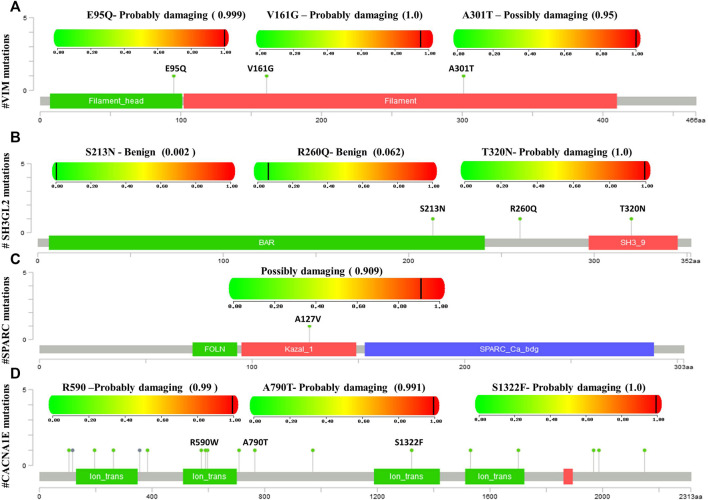
Lollipop plot exhibiting the significant substitution mutations of genes classified either as benign or damaging. **(A)** Significant mutations of *VIM*. **(B)** Significant mutations of *SH3GL2.* **(C)** Significant mutations of *SPARC.* **(D)** Significant mutations of *CACNA1E.*

Mutational analysis of the hub genes was inferred from the six TCGA datasets using cBioportal with a total of 1,893 samples. *SH3GL2* had the highest altered frequency (2.2%) with six missense (shallow deletion) mutations. These mutations were classified as variants of unknown significance (VUS). *BRSK1* had six VUS mutations with five missense and one truncating mutation with a frequency of 1.2% alterations. *MCM3* had ten VUS mutations with six missense and one truncating mutation. *MAPK8IP2* and *PPP2R2C* had two VUS mutations. The detailed mutation data of the hub genes are presented in Table 3. Genes *HSPG2* with 35 VUS had a higher number of mutations with 1.5% somatic mutations followed by *ASPM* and *CACNE1* with 26 VUS each. The substitution polymorphism was predicted using the PolyPhen-2 tool. It predicted the damaging effects of the hub genes. *VIM* had two mutations with probably damaging effects on V161G and E95Q with scores of 1.0 and 0.980, respectively. A possibly damaging effect with a score of 0.95 was also found on *A301T. DDN*, *MCM3*, and *MCTP1* had no mutations, while *SPARC* had only one mutation, A127V, which was possibly damaged with a score of 0.909. *SH3GL2* had only three mutations, out of which two were benign and the third where T was replaced by N at position 320 was probably damaging. *BRSK1* had four mutations, out of which G to A substitution that occurred at position 327 was probably damaging. *CACNA1E* had 22 mutations R to W substitution at 590th residue was found recurrent in many samples. The significant substitutions of the biomarker genes are represented in Figure 6. The genes *ASPM* and *HSPG2* exhibited 27 and 35 mutations, respectively. Some of their recurrent mutations are shown in Table 4. Figures 6A–D represents the significant substitution mutations of the four predominant genes classified as predictive biomarkers—*VIM*, *SH3GL2*, *SPARC*, and *CACNA1E*.

**TABLE 4:** Mutational analysis of the hub genes.

Gene	Somatic mutation frequency (%)	No. of VUS	No of missense	No. of truncating	No. of inframe/splice	Significant substitutions	Damaging effect	Score
*ASPM*-assembly factor for spindle microtubules	0.7	26	20	5	1	T2339P	Probably damaging	0.999
						S270P	Benign	0
						A3137D	Probably damaging	1.00
*HSPG2*-heparan sulfate proteoglycan	1.5	35	33	1	—	A1766D	Possibly damaging	0.623
						A2856T	Possibly damaging	0.926
*CACNA1E*-calcium voltage-gated channel subunit alpha1 E	1.0	26	25	1	—	S1322F	Probably damaging	0.99
						A709T	Probably damaging	0.991
						R590W	Probably damaging	1.0
*SH3GL2-*SH3 domain-containing GRB2-like 2, endophilin A1	0.3	6	6	0	0	S213N	Benign	0.002
						R260Q	Benign	0.062
						T320N	Probably damaging	0.093
*SPARC-*secreted protein acidic and rich in cysteine	<0.1	1	1	0	0	A127V	Possibly damaging	0.909
*VIM-*vimentin	0.1	3	3	0	0	V161G	Probably damaging	1.00
						A301T	Possibly damaging	0.95
						E95Q	Probably damaging	0.980
*MAPK8IP2*-mitogen-activated protein kinase 8 interacting protein 2	0.1	3	2	1	0	P499L	Benign	0.001
*BRSK1*-BR serine/threonine kinase 1	0.3	7	6	1	0	G327A	Probably damaging	1
						R418*	Probably damaging	0.999
						F136L	NA	—
						K135Q	NA	—
*PPI5K1*-diphosphoinositol pentakisphosphate kinase 1	<0.1	1	1	0	0	NA	—	—
*PPP2R2C-*protein phosphatase 2 regulatory subunit B'gamma	0.1	2	2	0	0	E128K	Benign	0.006
						R274H	Benign	0.013
*MCM3*-assembly factor for spindle microtubules	0.5	12	6	6	0	NA	—	—
*SYNJ2*-synaptojanin 2	0.5	13	12	1	0	P812S	Benign	0.160
						R376H	Probably damaging	1.00
*MCTP1-*multiple C2 and transmembrane domain-containing 1	0.4	9	2	4	3	NA		
*SV2B-*synaptic vesicle glycoprotein 2B	0.3	10	8	1	1	L294M	Probably damaging	1.0
						K295R	Benign	0.01
						A438T	Benign	0.535

### Survival Analysis

**FIGURE 7:**
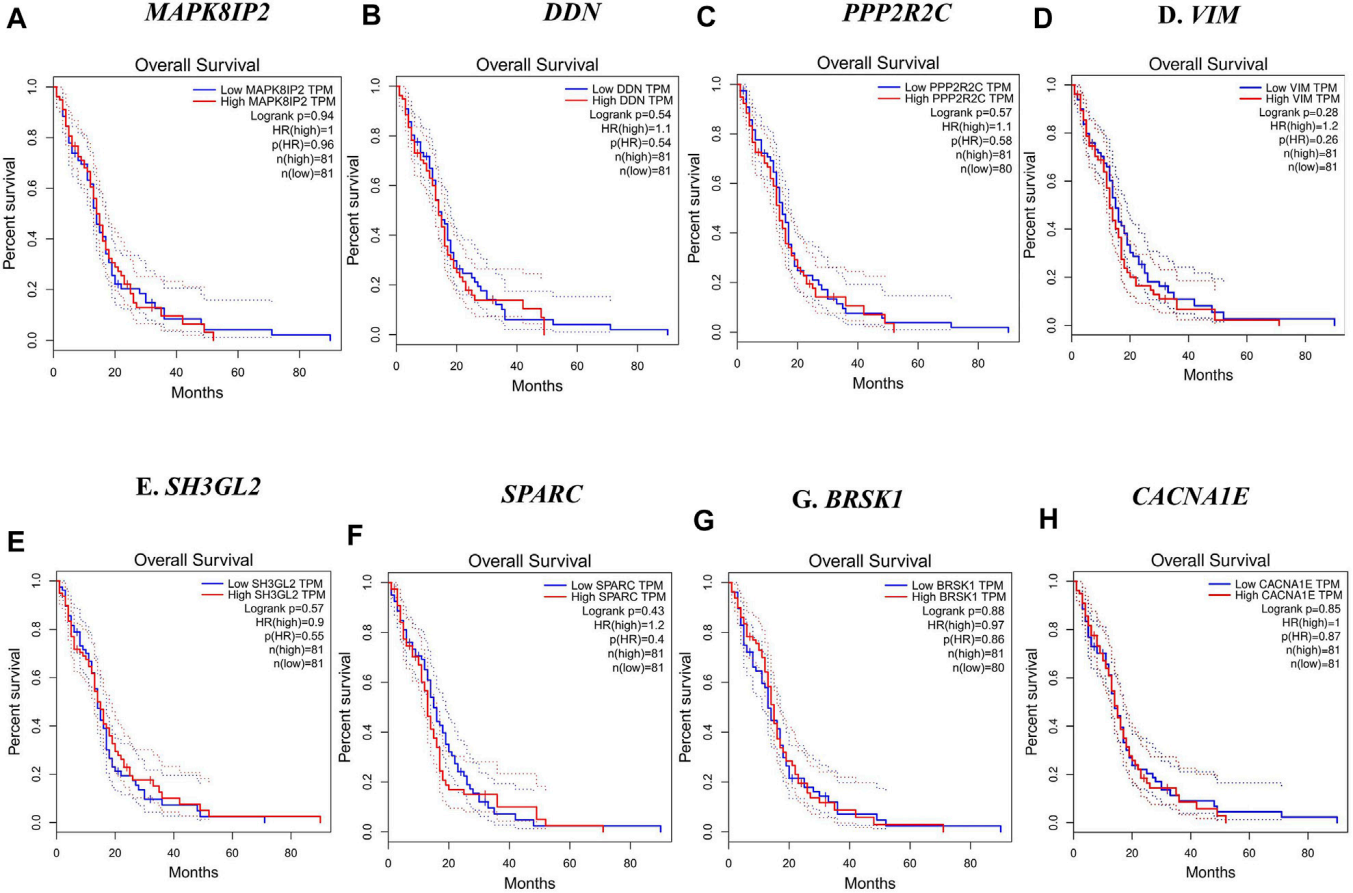
Overall survival analysis by Kaplan–Meir plots of the hub genes. **(A)** Overall survival rate of *MAPK8IP2* in GBM patients. **(B)** Overall survival rate of *DDN* in GBM patients. **(C)** Overall survival rate of *PPP2R2C* in GBM patients. **(D)** Overall survival rate of *VIM* in GBM patients. **(E)** Overall survival rate of *SH3GL2* in GBM patients. **(F)** Overall survival rate of *SPARC* in GBM patients. **(G)** Overall survival rate of *BRSK1* in GBM patients. **(H)** Overall survival rate of *CACNA1E* in GBM patients.

In this step, the prognostic benefits of the hub genes were explored in correlation with the overall survival rates of GBM patients. The median months of deceased and disease-free progression rates correlated with the hub genes expression (Table 5). A total of nine genes, namely, *DDN*, *SH3GL2*, *PPP2R2C*, *MAPK8IP2*, *SPARC*, *CACNA1E*, *VIM*, *MCM3*, and *BRSK1*, were identified to have an overall survival of more than 5. Log-rank *p*-value is only the test of significance based on the time of events. The overexpressed gene *SPARC* had an overall survival rate of 7.26 median months and the disease-free rate of two months which shows the progression of the disease. The underexpressed genes *MAPK8IP2*, *BRSK1*, *SH3GL2*, *DDN*, and *PPP2R2C* had overall survival rates of 10.65, 16.93, 6.54, 7.40, and 17.80 median months, respectively. The disease-free progression rates of *MAPK8IP2*, *BRSK1*, *SH3GL2*, *DDN*, and *PPP2R2C* were 11.17, 8.40, 13.21, 4.83, and 10.90 median months, respectively. The overall and disease-free progression of upregulated hub gene *VIM* had shown log*-p* ranks of 0.28 and 0.04, respectively. The highest overall survival rate was 65.33 median months. The highest disease-free progression rate was 57.72 median months. With five years of life expectancy, these values indicate a good survival rate and can be proposed as a prognostic biomarker for GBM. Figure 7 shows the overall survival rates of eight significant genes—*MAPK8IP2* (Figure 7A), *DDN* (Figure 7B), *PPP2R2C* (Figure 7C), *VIM* (Figure 7D), *SH3GL2* (Figure 7E), *SPARC* (Figure 7F), *BRSK1* (Figure 7G), and *CACNA1E* (Figure 7H).

**TABLE 5:** Survival analysis of the hub genes.

Gene	Molecular subtype	Mutual exclusivity or co-occurrence	Hazards ratio	Overall survival				Disease-free survival			
				Log *p-*rank	Number of cases	Number of deceased cases	Median months^a^	Log p-rank	Number of cases	Number of recurred cases	Median months
*DDN*	Upregulated—proneural	No significant co-occurrence or mutual exclusivity	1.08	0.54	13	10	7.40 (6.02–NA)	0.3	8	5	4.83 (4.80–NA)
	Downregulated—classical										
*SH3GL2*	Upregulated—proneural	Co-occurrence with SYNJ2	1	0.57	43	41	6.54 (5.39–19.80)	0.73	22	14	13.21 (8.50 -NA)
	Downregulated—classical										
*CACNA1E*	Upregulated—mesenchymal	No significant co-occurrence or mutual exclusivity	1.01	0.77	118	97	13.00 (7.39–15.70)	0.81	63	44	11.17 (8.02–14.98)
	Downregulated—proneural										
*PPP2R2C*	NA	No significant co-occurrence or mutual exclusivity	NA	0.57	10	7	17.80 (17.77–NA)	0.23	6	4	10.90 (10.87–NA)
*MAPK8IP2*	Upregulated—proneural	No significant co-occurrence or mutual exclusivity	0.63	0.94	32	29	10.65 (4.73–15.70)	0.34	15	10	11.17 (3.91–NA)
	Downregulated—mesenchymal										
*BRSK1*	NA	No significant co-occurrence or mutual exclusivity	NA	0.88	15	10	16.93 (16.90–NA)	0.44	8	4	8.40 (5.65–NA)
*SPARC*	Upregulated—classical	No significant co-occurrence or mutual exclusivity	0.86	0.43	5	5	7.26 (7.23–NA)	0.61	4	4	2.00 (2.00–NA)
	Downregulated—proneural										
*VIM*	Upregulated—classical	No significant co-occurrence or mutual exclusivity	1.07	0.28	12	7	65.30 (26.33–NA)	0.04	5	5	57.72 (50.70–NA)
	Downregulated—proneural										
*MCM3*	Upregulated—proneural	Co-occurrence with MCTP1 and PPP2R2C	0.82	0.89	9	2	NA	0.086	4	2	12.16 (12.16–NA)
	Downregulated—mesenchymal										

a NA: Data were not available to report.

### Prediction of Prognostic Potentials of Hub Genes Across GBM Subtypes

The GBM can be classified into three subtypes, namely, proneural, classical, and mesenchymal, based on their prognosis and survival rates. The factors such as inter tumor, intratumor heterogeneity, short survival, and lack of treatment contribute to this classification. According to Verhaak et al. (2010), proneural subtypes are found in less pathological conditions and young patients with better survival and prognosis rate. Recurrent GBM with a high incidence of the tumor, inflammation, and necrosis shifts to mesenchymal types and is considered the worst of all. The classical subtype showed various amplifications, including chromosome 7, deletion of chromosome 10, and lack of p53 mutations. Upon combative radiotherapy and chemotherapy, classical types had a reduced mortality rate (Zhang P et al., 2020). GBM Biodp database enabled the identification of prognostic potentials of the hub genes across the GBM subtypes and their expression levels are represented in Supplementary Figure S1. *DDN* was identified to be upregulated in the proneural subtype and downregulated in the classical subtype (Sidaway, 2017), while *SH3GL2* was upregulated in proneural and downregulated in classical, thus making them good prognostic biomarker candidates. *CACNA1E* with a hazards ratio of 1.01 was found upregulated in mesenchymal but downregulated in proneural. *SPARC* with a hazards ratio of 0.86 was observed in higher levels only during the later stages of GBM. They tend to be upregulated in classical and mesenchymal while exhibiting downregulated expressions in proneural. VIM had a higher hazards ratio of 1.07 and was observed upregulated in classical and downregulated in proneural. Both *MCM3* and *MAPK8IP2* were upregulated in proneural and downregulated in mesenchymal with the potential to act as prognostic biomarkers. When assessing their significant mutual exclusivity and co-occurrence ability filtered with a *p*-value <0.05, it was found that most of the genes including *DDN*, *SPARC*, *VIM*, *BRSK1*, *PPP2R2C*, and *MAPK8IP2* were free from mutual exclusivity and co-occurrence. *MCM3* was found to be co-occurring with *MCTP1* and *PPP2R2C*, while *SH3GL2* co-occurred with *SYNJ2*. Hence based on the classification of genes at the proneural type four genes, namely, *SH3GL2*, *MCM3*, *MAPK8IP2*, and *DDN*, have the potential to be suitable biomarker candidates.

### Identification of Prognostic Biomarkers Through Pan-Cancer Analysis

**FIGURE 8:**
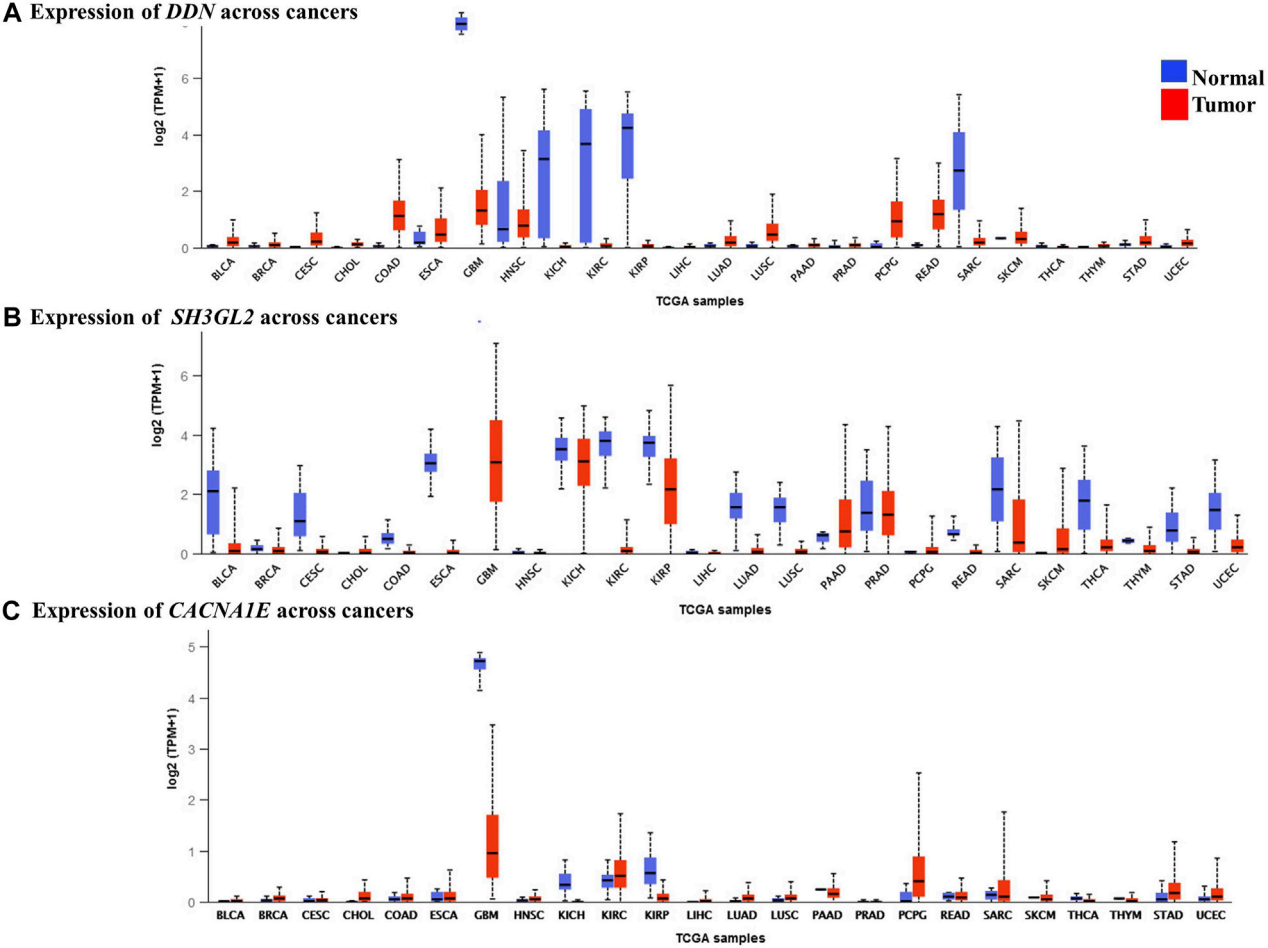
Expression levels of the hub genes in pan-cancer tissues. **(A)** Lower expression levels of *DDN* observed in GBM. **(B)** Lower expression levels of *SH3GL2* observed in GBM. **(C)** Lower expression levels of *CACNA1E* observed in GBM.

We performed a pan-cancer analysis to ascertain the occurrence and expression patterns of hub genes in normal and GBM tissues in comparison to other cancer tissues. Although the *SPARC* and *VIM* genes had higher expression rates in GBM, they were found high in other cancer types. In GBM, they can be considered as significantly overexpressed genes. Their upregulation in the mesenchymal and classical subtypes reveals that they are involved in the more aggressive stages of GBM. Four genes, namely, *MAPK8IP2*, *DDN*, *CACNA1E*, and *SH3GL2*, were significant, with higher expression rates of 345.24 TPM, 7.93 TPM, 21.65 TPM, and 7.92 TPM, respectively, in the normal brain and were significantly lower in GBM with respective expression rates of 24.28 TPM, 1.313 TPM, 0.8 TPM, and 3.06 TPM. In contrast, *MCM3* was found to be lowest in the normal brain with 16.09 TPM with a higher expression level of 121.564 TPM. Although *MCM3* was upregulated in proneural, it had exceptionally higher levels in GBM than in normal brains. It cannot be ascertained as a biomarker for GBM due to its similar higher expression patterns in various cancers. However, *MAPK8IP2* was significantly downregulated in GBM, lower brain grade glioma, and paraganglioma, as compared to the normal brain tissues. *MAPK8IP2* also loses its importance as a biomarker for GBM as it is also observed in all other cancer types. The highest expression level of *DDN* was seen in the normal brain tissues and a distinguishable reduction was observed in GBM. Similar lower levels of DDN were observed only in pheochromocytoma paraganglioma (PCPG), head and neck squamous carcinoma (HNSC), esophageal carcinoma (ESCA), colon adenocarcinoma (COAD), and rectal adenocarcinoma (READ). Apart from GBM, *SH3GL2* deviation was marked in the kidney chromophobe tumor (KICH), kidney renal cell carcinoma (KIRC), pancreatic adenocarcinoma (PAAD), prostate adenocarcinoma (PRAD), and sarcoma (SARC). Similar to *DDN* and *MAPK8IP2*, only the normal brain tissues had the highest expression of *CACNA1E* with a marked reduction of expressions in GBM. Lower expression levels of *CACNA1E* were found in pheochromocytoma, paraganglioma (PCPG), kidney renal clear cell carcinoma (KIRC), and sarcoma (SARC). Interestingly, due to less common occurrence in other cancer types, larger differences in expression rates in GBM than the normal tissue three genes, namely, *CACNA1E*, *DDN*, and *SH3GL2*, Figure 8 can be proposed as potential prognostic biomarkers of GBM. Although the survival rates of patients with *DDN* and *SH3GL2* were lower, they can still be considered putative diagnostic markers in GBM. This is due to the fact that they demonstrated a significant drop in expression rate as compared to the normal brain tissues. Significant overexpression of *VIM* with high overall survival correlated with previous findings as a strong biomarker of the mesenchymal and classical types of GBM (Shai et al., 2003).

## Discussion

GBM is the deadliest type of brain tumor. Unfortunately, to date, both the diagnosis and treatment are extremely challenging. In its aggressive form, the blood–brain barrier is disrupted, which worsens the delivery of oncogenic drugs and treatment (Hoelzinger et al., 2005). Elucidation of the molecular biomarkers and pivotal pathways assist in the early detection of the disease progression. In this aspect, computational gene profiling strategies have been advocated for GBM and other cancer types (Piao et al., 2021). In the current study, we have conducted an extensive investigation of the underlying gene expressions in GBM with an aim to identify the key candidate genes and molecular drivers responsible for their progression. Using integrated bioinformatics analysis, we have identified 61 differentially expressed genes (23 upregulated and 38 downregulated genes) by comparing the microarray expression profiles- GSE90604, GSE50161, and GSE134470, obtained from the NCBI-GEO database.

In the subsequent gene ontology analysis, we have identified that the voltage-gated ion channels of calcium, neurotransmitter receptor activities, cell communication, neuronal growth, synaptic transmission, and GABA receptor functions were disrupted in the GBM. Furthermore, the overexpressed immune responses, including interleukin, interferon, and cytokines were significant in GBM along with enhanced DNA replication. The pathway enrichment determined by KEGG and REACTOME for the annotated genes reveals the inter-relationships, interactions, and regulation of how each gene affects the other. Pathways overexpressed by the upregulated DEGs were cell cycle checkpoints, mitotic G1-G1/S phase, DNA replication, immune system signaling of interferons, interleukins, and cytokines, while the downregulated pathways were GABAergic and dopaminergic synaptic transmission, calcium signaling, osteoclast formation, SNARE formation, and methionine salvage pathways.

The detailed functional annotation revealed that the upregulation of DEGs such as *HSPG2*, *SOCS3*, *DDX58*, and *CLEC7A* was involved in inflammatory response—cytokine, interleukin 6 and 8, and interferon signaling. *MCM3* and *HELLS* responsible for the initiation of DNA replication, replication assembly complex, DNA methylation, and alkylation were enriched, which signifies the escalation of cell proliferation. The rise in cell morphogenesis and endothelial and epithelial cell proliferation was remarkably seen with the upregulated *SPARC* gene. The intermediate filament vimentin shot up the cellular response to muramyl dipeptide and regulation of collagen metabolism. A surge in neuronal mechanisms such as glial cell differentiation, axon extension, and regulation of myelination and neural precursor cell proliferation was seen due to the enrichment of *VIM*, *SEMA5A*, and *PTPRZ1.* In low-grade glioma, *SEMA5A* was underexpressed, exhibiting its tumor suppressor nature, but it is compromised in high-grade GBM (X. Li et al., 2012). Remarkable elevation of oligodendrocyte differentiation and angiogenesis were observed in GBM. Functional annotation unraveled the dwindling mechanisms of synaptic transmission, axodendritic transport, neuronal differentiation, postsynaptic membrane potential, transmembrane transport activity, and neuronal plasticity due to the underexpression of *DLG2*, *BRSK1*, *MAPK8IP2*, *SHISA7*, *SV2B*, and *CACNA1E*. All the downregulated DEGs were related to curtailment of brain development, neuronal transport, and synaptic transmission signals. *SV2B* was found to be differentially expressed in glioma grade II. Both over and underexpression of the gene were previously noted in GBM (Zhang Y et al., 2020). RNA polymerase II cis-regulatory region sequence-specific DNA binding, cognitive function, cellular response to brain-derived neurotrophic factor stimulus, neuron projection development, plasma membrane bounded cell projection organization, and nervous system development were declined with the reduction of their responsible genes—*DDN* and *SH3GL2*. The most remarkable pathway of the brain, the phosphatidylinositol biosynthetic process involved in aging, was downregulated due to the phosphatidylinositol 3-kinase (PI3K) dysfunction as seen in many neurodegenerative processes (Thakur & Rattan, 2012).

Protein–protein interaction profiles of the overlapped DEGs with a high confidence score unraveled interesting upregulated and downregulated protein interactions. The 11 upregulated hub genes were identified as *CTSK*, *HSPG2*, *SPARC*, *VIM*, *SOCS3*, *HELLS*, *CKAP2*, *ASPM*, *MCM3*, *DDX58*, and *CLEC7A.* Based on their PPI network, 16 downregulated hub genes viz. *MAPK8IP2*, *CACNA1E*, *BZRAP1*, *RIMS3*, *GABRD*, *DDN*, *BRSK1*, *NAP1L2*, *PPIP5K1*, *RASL10B*, *PPP2R2C*, *SYNJ2*, *SH3GL2*, *LICAM*, *MCTP1*, and *SV2B* were identified. Among them, seven upregulated DEGs— *VIM*, *HELLS*, *HSPG2*, *MCM3*, *ASPM*, *SPARC*, and *SOCS3* and three downregulated DEGs—*SH3GL2*, *LICAM*, and *SYNJ2* exhibited interactions with a high confidence score of 0.7. All the hub genes were validated by comparing their expression patterns in the GBM datasets of TCGA from which the genes *PPP2R2C*, *DDN*, *SH3GL2*, *MAPK8IP2*, *CACNA1E*, and *BRSK1* were found significantly underexpressed, whereas *SPARC*, *VIM*, and *MCM3* were highly expressed in both the TCGA datasets (including the TP53 mutant and non-mutant types) and GEO datasets. On the contrary, the genes *CTSK*, *CKAP2*, *DDX58*, and *HSPG2* of GBM in TCGA were observed to be significantly overexpressed in comparison to their expressions in GEO datasets. The genes *SYNJ2* and *RIMS3* were observed to be relatively underexpressed in the TCGA datasets to the GEO datasets. We were also interested in predicting the driver or passenger mutations for the hub genes. We found *HSPG2* with 35 VUS had the higher number of mutations followed by *ASPM* and *CACNA1E* with 26 mutations each. The mutational frequency of the three genes demands more insight and analysis to be identified as marker genes. *CACNA1E*, the member of voltage-gated calcium channels, was identified as a significantly downregulated gene and was identified to be one of the unique genes of GBM (Phan et al., 2017). Predominant substitution mutations of the hub genes were inferred to understand if they are benign or damaging.

Furthermore, we have conducted a survival analysis to identify the putative biomarkers of GBM, and a similar approach for biomarker identification has been reported in other studies (Gulluoglu et al., 2018; Gulluoglu et al., 2018; Piao et al., 2021). The overall survival and disease-free survival analysis narrowed down the initially identified hub genes to eight noteworthy genes, namely, *SH3GL2*, *SPARC*, *MAPK8IP2*, *DDN*, *BRSK1*, *CACNA1E*, *VIM*, and *PPP2R2C*, whose overall survival rate was greater than five months. *VIM*, with a log-rank *p*-value of 0.28, had a better life expectancy of more than five years and their expression levels are strikingly high in GBM.

The most interesting findings of this study are the identification of potential biomarkers in GBM. Genes, namely, *PPP2R2C* (protein phosphatase regulatory subunit B gamma), *SH3GL2* (SH3 domain-containing GRB2-like 2, endophilin A1), *BRSK1* (BR serine/threonine kinase 1), *DDN* (dendrin), *CACNA1E* (calcium voltage-gated channel subunit alpha1 E), and *MAPK8IP2* (mitogen-activated protein kinase 8 interacting protein 2) were harmoniously underexpressed in GBM than in the normal tissues. These genes were also observed to be significantly downregulated in GBM, as indicated by the pan-cancer analysis. Interestingly, as per GeneCards, the *PPP2R2C* gene was already reported to be involved in cell cycle regulation, beta-adrenergic receptor signaling, and PI3-Akt signaling, as well as being responsible for inflammatory bowel disease (Lizcano et al., 2004). *MAPK8IP2* was responsible for AKT, ERK, and MAP signaling pathways and was responsible for spinocerebellar ataxia (Ziats et al., 2019). *DDN* is a significant gene validated to have a role in causing autism with properties to heal impaired bone density (Cousminer et al., 2018). *BRSK1* was reported to be a biomarker for lung large cell carcinoma with a role in LKB1 signaling (Lizcano et al., 2004)). *SH3GL2* exhibited an important role in clathrin-mediated endocytosis and its underexpression caused pediatric pilocytic astrocytoma (Yao et al., 2014). Hence, the lower expression levels of the above said five genes correlated with either disruption of signals or causing neurological distress. The two predominant upregulated hub genes, namely, *VIM* (vimentin) and *SPARC* (secreted protein acidic and rich in cysteine), showed significantly higher expression rates in GBM than in normal brains. Vimentin (*VIM*), the major intermediary filament constituent, was associated with neuritogenesis, cell signaling, attachment, and migration, causing congenital cataracts (Griesinger et al., 2013). There is ample evidence on *VIM* as a potential biomarker or therapeutic candidate for GBM. *VIM*, a multifunctional protein, exhibits interactions with diverse proteins and ascertains itself to be a marker for highly aggressive and metastatic forms of almost all cancers (Shai et al., 2003). The histochemical profiles of *VIM* revealed that it was widely distributed in gliomas, cerebellar pilocytic astrocytomas, neurinomas, and endothelial cells of various cancer cells (Schiffer et al., 1986). Higher expression of *VIM* was attributed to the progression of glioblastoma and was linked to a reduced survival rate (Zhao et al., 2018). Cell surface vimentin on the GBM has been shown to initiate tumors in the adjacent cells, and two monoclonal antibodies 86C and pritumumab were successful to target cell surface vimentin offering promising treatment options to GBM (Noh et al., 2016; Babic et al., 2018). *VIM* was seen upregulated in the classical and mesenchymal subtypes and evidently the transitions from classical to mesenchymal GBM were correlated to higher expressions of *VIM* (Schiffer et al., 1986; Herrera-Oropeza et al., 2021). Upregulation of vimentin protein was evident in the proliferation and migration of GBM. The invasion of cancer cells was found to be suppressed by vimentin knockdown strategies (Nowicki et al., 2019). Although pan-cancer analysis showed overexpression of *VIM* in most cancer types, its overall survival rate was promising, with 65.3 months of life expectancy in patients, thus making it an attractive diagnostic biomarker for aggressive stages of GBM. It is also due to the fact that it has a very high expression rate in mesenchymal and classical subtypes. *SPARC*, also termed osteonectin, was associated with brittle bone disorder with significance in metastasis and cancer invasion. It was found upregulated in mesenchymal and classical subtypes (Delany et al., 2008) due to which the overall survival rate of *SPARC* was just 7 months and disease-free survival was 2.2 median months. Similar to *VIM*, although *SPARC* was remarkably overexpressed in most cancer types, a ten-fold increased expression seen in GBM makes it crucial (Liu & Lathia, 2016); (Yao et al., 2014); (Herrera-Oropeza et al., 2021). The fact that the expression of *DDN* and *SH3GL2* were attributed to the proneural subtype makes them strong prognostic GBM biomarker candidates. *CACNA1E* was attributed to the mesenchymal subtype, which is the most aggressive form of GBM, thus making it a significant diagnostic biomarker for GBM. Furthermore, pan-cancer analysis of the hub genes revealed three genes, namely, *CACNA1E*, *DDN*, and *SH3GL2*, which were predominantly downregulated in GBM but not identified in more than five cancer types, could also make them putative prognostic biomarkers for GBM*.*

There is enough evidence on the prognostic biomarkers of GBM. Putative biomarkers for the GBM stem cells were identified: upregulation of *CD133* (encoded by *PROM1*) is linked to self-renewal of stem cells and resistance to temozolomide (Han et al., 2016), *CD44* is found to be involved in tumor cell migration and proliferation (Nishikawa et al., 2018), *CD15* (a trisaccharide 3-fucosyl-N-acetyllactosamine) as seen in many cancers is attributed to the GBM grades and survival during hypoxic conditions (Ishii et al., 2021), *CD70* (*CD27* L—type II transmembrane protein that belongs to the tumor necrosis factor (*TNF*) receptor family) is attributed to tumor immunosuppression and aggressiveness of GBM (Pratt et al., 2017), *S100A4* is a metastasis inducer capable of initiating a tumor and forming spheres of GBM (Liang et al., 2014), *ALDH1A3* (aldehyde dehydrogenases) is linked to tumor proliferation in multiple cancers (Fedele et al., 2019), nanog (homeodomain transcription factor) is linked to low survival in both low- and high-grade glioma (Elsir et al., 2014), *OCT-4* (octamer-binding protein transcription factor 4) is found to be upregulated in the hypoxic conditions of GBM (Krogh Petersen et al., 2016), *SOX-2* (sex-determining region Y) is found to have an increased expression in GBM stem cells linked to the growth of tumors and relapse after chemo and radiotherapy (Ren et al., 2016), and nestin (an intermediate filament) is attributed to tumor initiation, angiogenesis, metastasis, and aggressive growth (Nowak et al., 2018). Although several putative prognostic biomarkers for GBM are already predicted, they are found in multiple cancer types (Hassn Mesrati et al., 2020). Mutations on *IDH1* (isocitrate dehydrogenase) were considered significant with prognostic benefits, while deletion of *CDKN2A* (cyclin-dependent kinase inhibitor 2A) in IDH mutants was a marker for malignancy. *TERT* (telomerase and reverse transcriptase) promoter mutations, *H3F3A* (replication-independent histone 3.3 linked to high-grade gliomas) alterations, and methylation of *MGMT* (O6-methylguanine-DNA methyltransferase) promoters were proposed as potential markers of GBM (Śledzińska et al., 2021). The potential prognostic biomarkers, namely, epidermal growth factor (*EGFR*), p53 (tumor suppressor protein), platelet-derived growth factor receptor (*PDGFR*), phosphoinositide 3-kinase (PI3K), phosphatase and tensin homolog (*PTEN*), and 1p/19q (codeletion of chromosomes 1p and 19q) have also been identified, but they failed to achieve prognostic effect in the clinical studies (Karsy, 2015). Nanoparticle protein typing of the extracellular vesicles revealed the protein markers *EGFR* (epidermal growth factor receptor), *IDH1*, *PDPN* (podoplanin), *TGFB* (transforming growth factor-beta), *IL*-8 (interleukin 8), *TIMP1* (*TIMP* metallopeptidase inhibitor 1), and *ZAP70* (zeta chain-associated protein kinase 70). According to GBM subtype classification, chromosome 7 amplification together with the deletion of chromosome 10 and *EGFR* amplification, were identified as a classical GBM (Verhaak et al., 2010). Mesenchymal GBM showed mutations in neurofibromatosis type 1 (NF1) with the upregulation of necrosis and inflammation genes (Fadhlullah et al., 2019). Proneural subtype GBM was marked with *IDH1* point mutations and platelet-derived growth factor receptor alpha (*PDGFRA*) aberrations (Verhaak et al., 2010). Sequencing of the circulating tumor DNAs revealed the presence of mutations in *IDH1*, *IDH2*, *TP53*, *TERT*, *ATRX* (nuclear alpha-thalassemia/mental retardation X-linked syndrome), *H3F3A*, and *HIST1H3B* mutations claiming them to be significant biomarkers of GBM. The drawback of the predicted circulating tumor DNAs is that they are very rarely detected. *SH3GL2*, a tumor suppressor gene widely prevalent in the central nervous system, was identified to be downregulated by the miRNA biomarker—mir330, thereby causing malignancy in GBM (Yao et al., 2014). Although there were a remarkable number of biomarkers already reported, they either failed to exhibit prognostic effects in clinical studies or were invariably seen in many cancer types, making them futile.

Altogether, through this study, we provide sufficient background for the genes *SH3GL2*, *DDN*, and *CACNA1E* to be the potential putative prognostic biomarker candidates of GBM. *BRSK1*, *PPP2R2C*, and *MAPK8IP2* with striking lower expression levels and better survival rates can also play a role in the early diagnosis of GBM. The study evaluated the biomarkers in comparison with GEO datasets, TCGA datasets, and the Pan-Cancer Analysis of Whole Genomes datasets adding strong validation to the predicted biomarkers.

## Conclusion

This study sheds light on the identification of the key molecular drivers of GBM. The study elucidated putative prognostic biomarkers through a top–down integrated bioinformatics approach. Through this study, we have predicted novel GBM biomarkers *DDN* and *SH3GL2* along with the already reported *VIM*, *CACNA1E*, and *SPARC* genes. It provides a promising preliminary investigation that employs multiple steps of validation right from the comparison of expression levels between normal and GBM brain tissues to predicting the prognostic potential based on the GBM subtypes. Further biological validation will be more valuable. Also, the clinical examination of the hub genes will endorse the prognostic biomarker candidates obtained through this study.

## Data Availability

The original contributions presented in the study are included in the article/Supplementary Material, further inquiries can be directed to the corresponding author.

## Supplementary Material

Supplementary Figure S1 Box plots representing the expression levels of hub genes classified into the subtypes of GBM—proneural (P) in pink, classical (C) in blue, and mesenchymal (M) in red. **(A)** Expression of *DDN* in three aggregate datasets classified as C, M, and P. **(B)** Expression of *MAPK8IP2* in three aggregate datasets classified as C, M, and P. **(C)** Expression of *MCM3* in three aggregate datasets classified as C, M, and P. **(D)** Expression of *VIM* in three aggregate datasets classified as C, M, and P. **(E)** Expression of *CACNA1E* in three aggregate datasets classified as C, M, and P. **(F)** Expression of *SPARC* in three aggregate datasets classified as C, M, and P.
