# Supplementary figures and images for: Gene Expression Profiling of Glioblastoma to Recognize Potential Biomarker Candidates
Source: Front Genet. 2022 Apr 27;13:832742. doi: 10.3389/fgene.2022.832742 (PMC9091202; doi:10.3389/fgene.2022.832742)

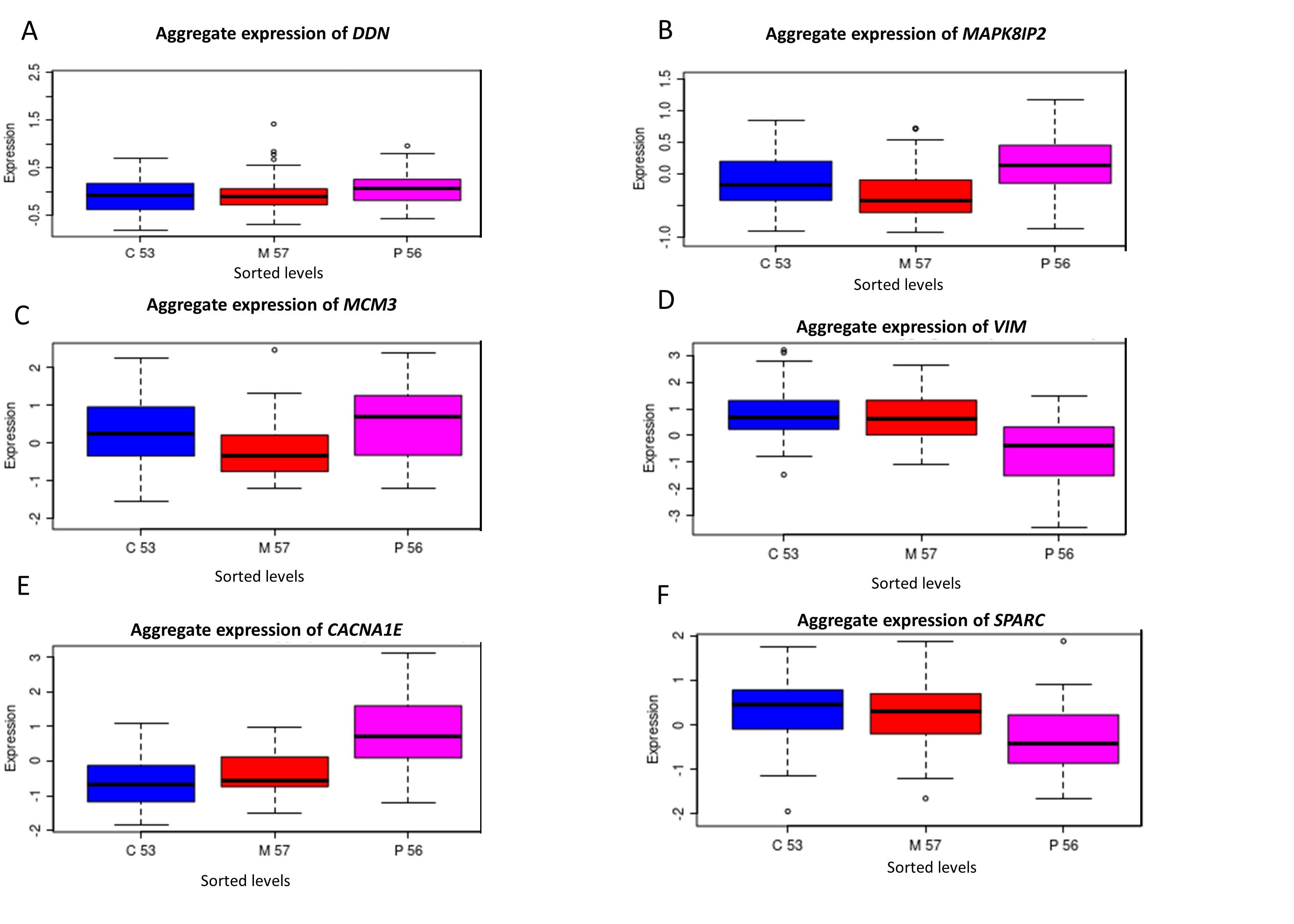

Supplement: Supplementary file 1 [file Image1.JPEG]
